# Hydrocephalus, massive myelitis, and adhesive arachnoiditis: full neuroaxis involvement by neurocryptococcosis

**DOI:** 10.1055/s-0043-1768159

**Published:** 2023-06-28

**Authors:** André Eduardo de Almeida Franzoi, Tamiris Maier Silva Ferreira, Emanuele Therezinha Schueda Stonoga, Bernardo Corrêa de Almeida Teixeira, Rosana Herminia Scola

**Affiliations:** 1 Universidade Federal do Paraná, Hospital de Clínicas, Serviço de Neurologia, Departamento de Clínica Médica, Curitiba PR, Brazil.; 2 Universidade Federal do Paraná, Hospital de Clínicas, Serviço de Infectologia, Departamento de Clínica Médica, Curitiba PR, Brazil.; 3 Universidade Federal do Paraná, Hospital de Clínicas, Serviço de Anatomia Patológica e Histopatologia, Departamento de Clínica Médica, Curitiba PR, Brazil.; 4 Universidade Federal do Paraná, Hospital de Clínicas, Serviço de Radiologia, Departamento de Clínica Médica, Curitiba PR, Brazil.; 5 Universidade Federal do Paraná, Hospital de Clínicas, Serviço de Doenças Neuromusculares, Departamento de Clínica Médica, Curitiba PR, Brazil.

**Figure 1:**
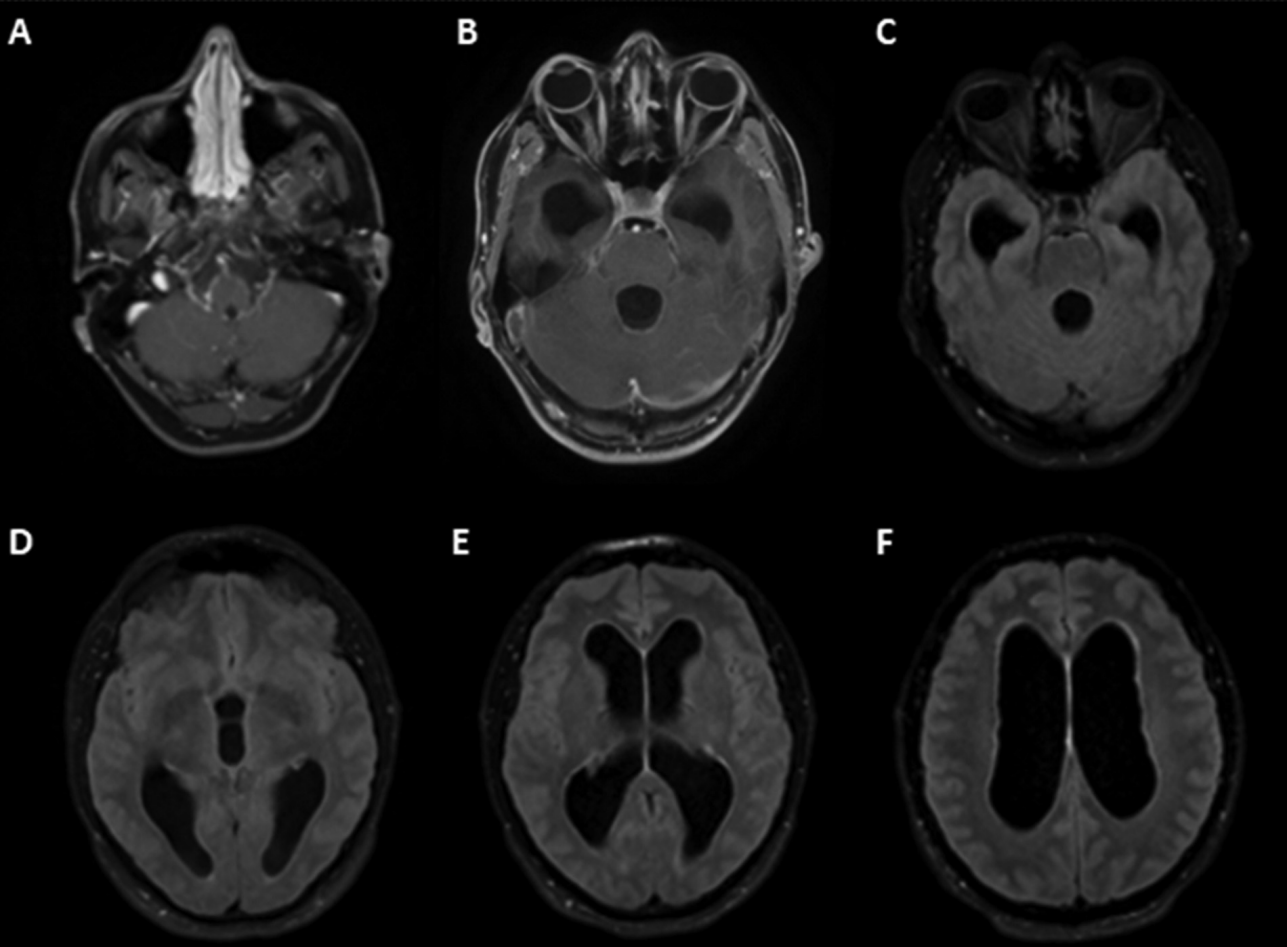
( **A,B** ) Axial contrast-enhanced T1-weighted magnetic resonance imaging (MRI) scan revealing leptomeningeal enhancement at the base of the brain in the posterior fossa; ( **C–F** ) axial fluid-attenuated inversion recovery (FLAIR) MRI showing hydrocephalus throughout the ventricular system, without significant transudation of the cerebrospinal fluid.

**Figure 2:**
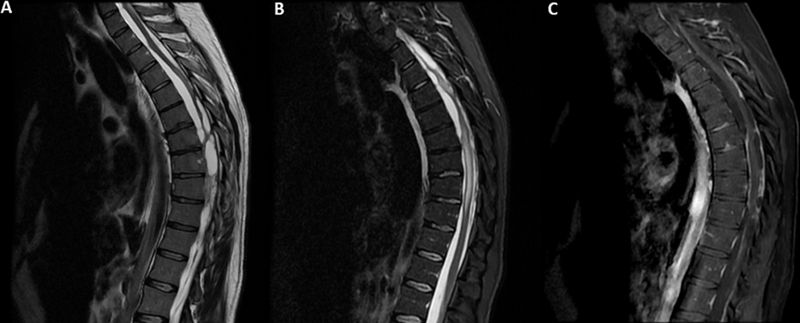
( **A** ) Sagittal T2-weighted MRI showing septations in the subarachnoid space around the spinal cord; ( **B** ) sagittal short-tau inversion recovery (STIR) MRI showing hyperintensity and distortion in the spinal cord; ( **C** ) sagittal contrast-enhanced T1-weighted MRI revealing leptomeningeal enhancement around the entire spinal canal.

**Figure 3:**
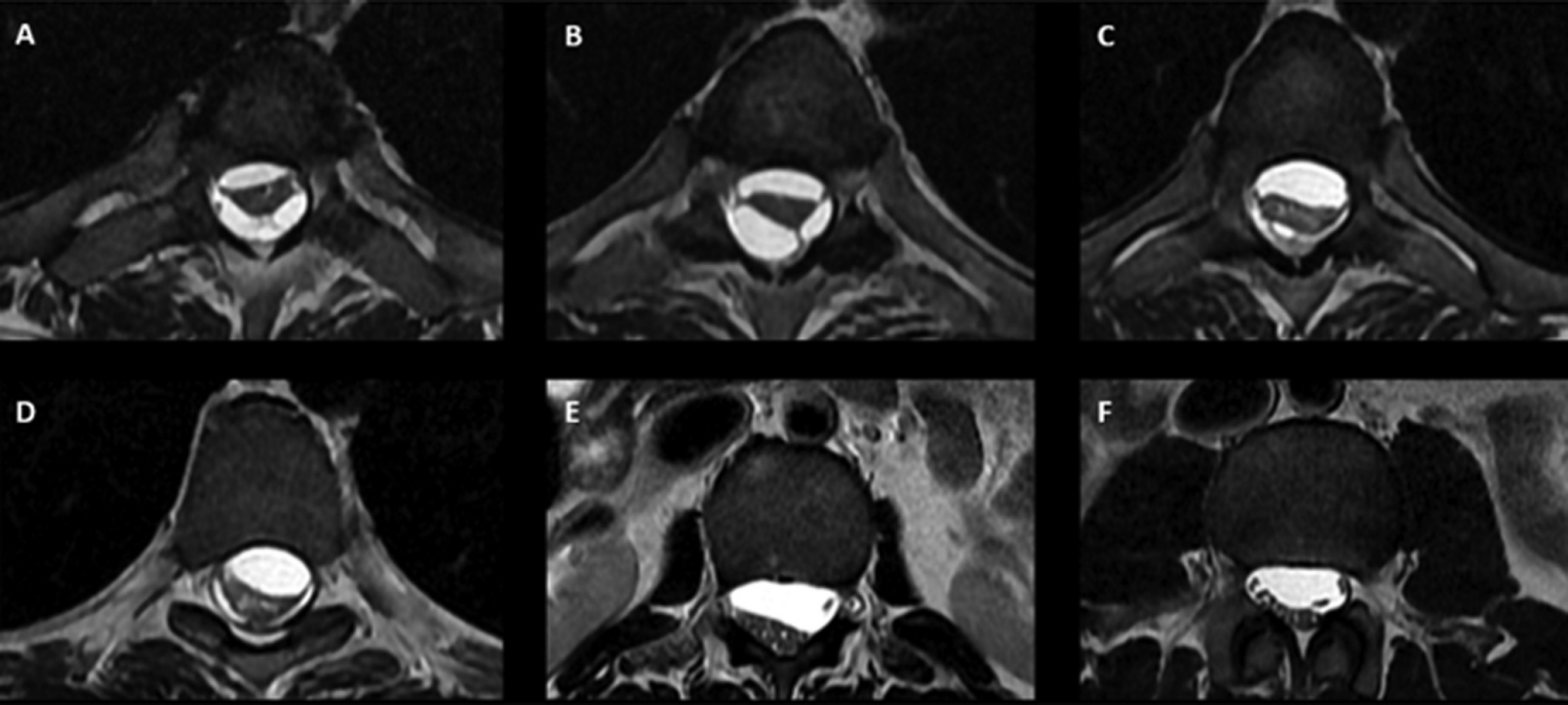
( **A–D** ): Axial T2-weighted MRI showing adhesive arachnoiditis and septations distorting the spinal cord at the level of the thoracic spinal cord; ( **E,F** ) axial T2-weighted MRI showing adhesive arachnoiditis and septations distorting the spinal cord at the level of the lumbosacral spinal cord.

**Figure 4:**
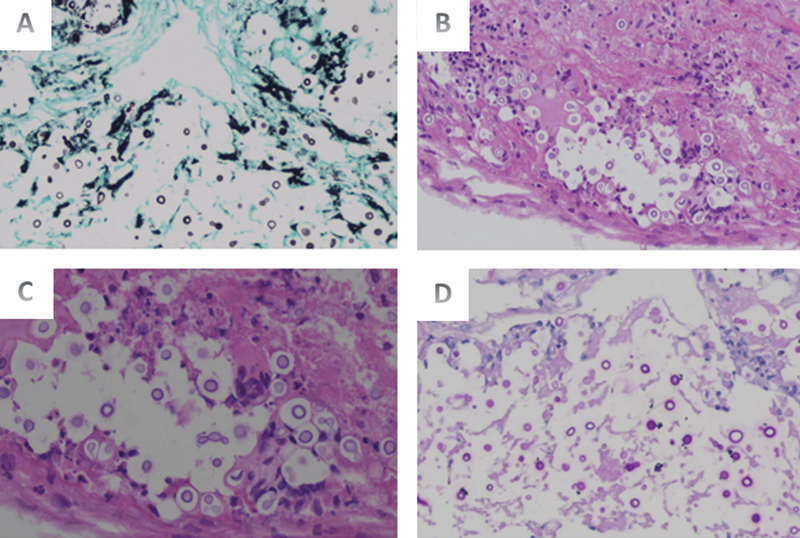
Encapsulated, spherical-to-oval yeast cells (5–10 μm in diameter) with narrow-based budding and polysaccharide capsules. The yeast cells vary in size, and the organisms can be capsule-deficient. ( **A** ) Grocott methenamine silver (GMS), smallest increase (×20); yeast cells tested positive for GMS; ( **B** ) hematoxylin and eosin staining, the smallest increase (×20); ( **C** ) periodic acid Schiff–diastase (PAS‒D), highest magnification (×40); yeast cells tested positive for PAS‒D staining; ( **D** ) PAS‒D, smallest increase (×20).

A 37-year-old male patient presented with subacute paraparesis, urinary incontinence, and a sensory level of T8. An analysis of the cerebrospinal fluid revealed lymphocytic pleocytosis (5 white blood cells/mm ^3^ ), low levels of glucose (25 mg/dL), increased levels of protein (713 mg/dL), high levels of lactic acid (4.7 mmol/L), and positive cryptococcal antigen. A magnetic resonance imaging (MRI) scan showed hydrocephalus ( Figure 1 ), myelopathy ( Figure 2 ), and adhesive arachnoiditis ( Figure 3 ). Meningeal biopsy showed round cells suggestive of cryptococcosis ( Figure 4 ), without species differentiation in the culture samples. *Cryptococcus* may exhibit unique clinical manifestations, such as gelatinous pseudocysts in the basal ganglia, cerebral cryptococcomas, leptomeningitis, cranial neuropathies, adhesive arachnoiditis, and obstructive hydrocephalus. 1 2 3 4 5
